# Acute Encephalitis Syndrome Surveillance, Kushinagar District, Uttar Pradesh, India, 2011–2012

**DOI:** 10.3201/eid1909.121855

**Published:** 2013-09

**Authors:** Manish Kakkar, Elizabeth T. Rogawski, Syed Shahid Abbas, Sanjay Chaturvedi, Tapan N. Dhole, Shaikh Shah Hossain, Sampath K. Krishnan

**Affiliations:** Public Health Foundation of India, New Delhi, India (M. Kakkar, E.T. Rogawski, S.S. Abbas);; University College of Medical Sciences, New Delhi (S. Chaturvedi);; Sanjay Gandhi Postgraduate Institute of Medical Sciences, Lucknow, India (T.N. Dhole);; Centers for Disease Control and Prevention, New Delhi (S.S. Hossain);; Office of the World Health Organization Representative to India, New Delhi (S.K. Krishnan)

**Keywords:** acute encephalitis syndrome, AES, Japanese encephalitis, Japanese encephalitis virus, JEV, surveillance, India, viruses, epidemiology, etiology

## Abstract

In India, quality surveillance for acute encephalitis syndrome (AES), including laboratory testing, is necessary for understanding the epidemiology and etiology of AES, planning interventions, and developing policy. We reviewed AES surveillance data for January 2011–June 2012 from Kushinagar District, Uttar Pradesh, India. Data were cleaned, incidence was determined, and demographic characteristics of cases and data quality were analyzed. A total of 812 AES case records were identified, of which 23% had illogical entries. AES incidence was highest among boys <6 years of age, and cases peaked during monsoon season. Records for laboratory results (available for Japanese encephalitis but not AES) and vaccination history were largely incomplete, so inferences about the epidemiology and etiology of AES could not be made. The low-quality AES/Japanese encephalitis surveillance data in this area provide little evidence to support development of prevention and control measures, estimate the effect of interventions, and avoid the waste of public health resources.

Acute encephalitis syndrome (AES) is a clinical condition caused by infection with Japanese encephalitis virus (JEV) or other infectious and noninfectious causes. A confirmed etiology is generally not required for the clinical management of AES. Thus, surveillance for JEV infection in India has focused on identifying AES cases rather than JE cases; this approach is more feasible given the limitations of public health resources (*1*). However, identification of the etiologic agent is necessary for planning relevant interventions. The standard for determining the etiology of AES is examination of cerebrospinal fluid (CSF) during the acute phase of illness; pathogen-specific IgM capture ELISA or nucleic acid amplification techniques are used to detect pathogens in the CSF. Serologic tests for pathogen-specific antibodies and virus detection in serum are also recommended. However, examination of CSF is preferred because serologic test results may indicate the presence of antibodies in the serum, but the AES may have a cause different than the agent producing the detected antibodies (*1*–*3*).

A good quality surveillance system with laboratory support is essential for understanding the causes of AES and responding appropriately. Accordingly, the National Vector Borne Diseases Control Programme in New Delhi, India, has developed guidelines for AES surveillance that promote the need for a strong surveillance system as a critical component for any control activities. In these guidelines, the goals outlined for AES surveillance are to 1) assess and characterize the burden of JE, 2) detect early warning signals for an outbreak, 3) assess the effect of vaccination, and 4) guide future strategies (*1*). The National Vector Borne Diseases Control Programme has also implemented several measures to strengthen local health systems, including building on the capacity of the health workforce to provide better clinical management, extending referral diagnostic facilities by upgrading the existing Baba Raghav Das (BRD) Medical College facilities and setting up a National Institute of Virology field unit; and establishing a dedicated surveillance unit in the Department of Preventive and Social Medicine at BRD Medical College to provide improved surveillance and outbreak responses (*4*).

From the 1970s until around 2010, JEV infection was considered to be the leading cause of AES in the traditional JE belt of India, which includes Kushinagar District in the state of Uttar Pradesh (*5*–*11*). However, because of a large number of JE cases of unknown etiology, AES patterns alone have not suggested a clear picture of the epidemiology of the disease. In recent years, despite of the introduction of a JE vaccine, an increased number of AES cases have been reported in India, including Uttar Pradesh, and the disease has spread to new districts, urban areas, and villages without pigs, which are not usually associated with JE transmission (*12**,**13*). Thus, the assertion that JEV is the leading cause of AES has been questioned, and other infectious agents, such as enteroviruses, have been reported as a cause of AES in Uttar Pradesh and other parts of India (*14*–*19*). A substantial contributor to the ambiguity about the etiology of AES could be the fact that surveillance data for AES have not been analyzed to assess reasons for the increased cases and other reported causes. We examined the completeness and quality of AES surveillance data from Kushinagar District, an area where JEV is highly endemic. Herein, we discuss the ability to make inferences about AES epidemiology and etiology from these data and the implications of our findings for policy planning and program implementation.

## Methods

### Source of Surveillance Data

The sentinel site for JE surveillance in Kushinagar District is the district hospital. Using a standard format, the hospital reports all AES/JE cases that meet the standard World Health Organization case definition (*3*) to the district malaria officer; this officer then forwards compiled data to the state program officer for transmission to National Vector Borne Disease Control Programme (http://nvbdcp.gov.in/Doc/AES%20guidelines.pdf). BRD Medical College in Gorakhpur, Uttar Pradesh, is a nearby regional field laboratory of the Indian Council of Medical Research, which receives clinical samples from patients admitted to the district hospital in Kushinagar and completes most laboratory testing for JEV infections. In addition, BRD Medical College serves as a tertiary care center for patients who directly seek medical care or who are referred from nearby districts, including Kushinagar. A line list with laboratory results of these patients is reported by BRD Medical College to the district malaria officer in Kushinagar for submission to state program officers.

In July 2012, we obtained the AES line lists data for January 2011–June 2012 that were submitted by BRD Medical College to Kushinagar District Headquarters in Padrauna. The list was originally to be used to identify blocks in Kushinagar District with high, medium, and low numbers of AES cases so that sites could be selected for a larger study of the drivers of JEV transmission. Cases recorded in the line lists represented case-patients from Kushinagar District who were 1) admitted directly to BRD Medical College, 2) referred to BRD Medical College by the district hospital in Padrauna, and 3) admitted to the district hospital in Padrauna but had serum samples referred to BRD Medical College because no diagnostic kits were available and laboratory testing was limited at the district hospital. An individual AES patient tracking system does not exist in the state of Uttar Pradesh, so patients treated in the private sector or at the district hospital in Gorakhpur were not included in the surveillance lists.

### Preparation of Surveillance Database for Analysis

Individual identifiers for the case-patients were removed from the line lists to ensure confidentiality. Data were cleaned (i.e., extraneous data were removed) in multiple steps, resulting in 4 changes being made to the AES line lists. First, the spelling of residential localities (block, village, or police station) were matched and standardized to result in a list of 32 residential areas. Residential areas were then categorized and combined by block (14 blocks in Kushinagar). Modifications were validated by cross-checking with village population lists provided by Savera, a local nongovernmental organization. Second, all age values were standardized to a uniform decimal system (e.g., 1 year and 6 months was changed to 1.5 years, and 8 months was changed to 0.67 year). Third, the dates of symptom onset, hospital admission, sample collection, and outcome (i.e., discharged, left against medical advice, or death) were standardized to 1 format. In the original list, dates were variously coded by using different notations (e.g., 4 March 2011 was mentioned within the same row as both 04/03/11 and 03/04/11). Assuming that the case data were entered in chronological order, we standardized dates by using, as a cue, the dates of the preceding and following AES cases. Fourth, case-patients who died, were absent, or left against medical advice were considered to have been discharged.

### Data Analysis

Cleaned data were imported into SAS version 9.2.2 (SAS Institute, Inc., Cary, NC, USA) for analysis. AES cases were plotted by week and month by using the recorded dates of symptom onset. We tabulated demographic characteristics of AES case-patients for 2011 and 2012 and stratified case-patients by block, vaccination status, laboratory test result for JEV infection, and clinical outcome. Incidence for 2011 was calculated by using population denominators from the 2011 Census of India (*20*). We calculated incidence overall, by sex, and for children 0–6 years of age by using the age stratification available in the district-level census data. We estimated the incidence for each block by using block-specific population denominators projected with the decadal growth rate from the 2001 Census of India (*20*). Crude incidence rate ratios were estimated with Wald-based CIs.

We also determined the median number of days between key points in AES disease progression and diagnosis: time between onset of symptoms and hospital admission, onset of symptoms and serum sample collection, hospital admission and serum sample collection, and hospital admission and discharge or death. Using previously described methods (*21*), we evaluated the quality of the surveillance data by assessing the amount of data cleaning required, the proportion of missing or incomplete values in line list fields, and inconsistencies in dates recorded for key points in AES disease progression and diagnosis.

## Results

### AES Epidemiology

**Figure 1:**
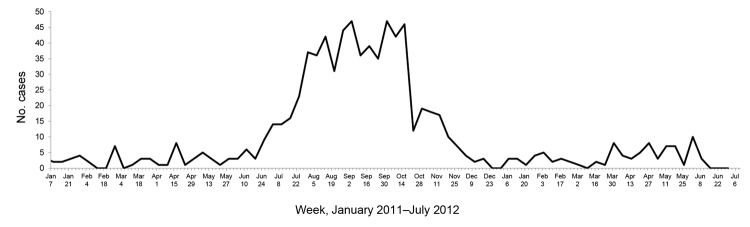
Weekly number of acute encephalitis syndrome cases, by month, in Kushinagar District, Uttar Pradesh State, India, 2011–2012. Numbers are based on data obtained from Baba Raghav Das Medical College, Gorakhpur, Uttar Pradesh, India.

In 2011, a total of 721 AES cases from Kushinagar District were identified through BRD Medical College; in 2012 (January–June), 91 cases were identified. Using the cleaned line lists, we determined the weekly number of AES cases reported during January 2011–June 2012 (Figure 1). Cases peaked during August–October 2011; >150 cases were identified in each of these 3 months. This seasonal trend corresponds with an expected increase in cases during the monsoon season, when transmission of both waterborne and vector-borne diseases increases (vector density is at its maximum).

In 2011 and 2012, most case-patients were male (57.4% and 59.3%, respectively) (Table 1). In 2011, almost half of the AES cases were in children <5 years of age (44.7%); the distribution of cases by age group was not substantially different in 2012. The case-fatality rate was 18.0% in 2011 and 19.8% in 2012.

**Table 1:** Characteristics of case-patients with acute encephalitis syndrome, Kushinagar District, Uttar Pradesh, India, 2011–2012*

Characteristic	No. (%) cases	
	2011, n = 721	2012, n = 91
Age, y		
0–4	322 (44.7)	36 (39.6)
5–9	205 (28.4)	22 (24.2)
10–14	83 (11.5)	12 (13.2)
>15	111 (15.4)	21 (23.1)
Sex		
M	414 (57.4)	54 (59.3)
F	307 (42.6)	37 (40.7)
Religion		
Hindu	632 (87.7)	80 (87.9)
Muslim	89 (12.3)	11 (12.1)
Vaccinated against JEV		
Yes	3 (0.4)	0
No	116 (16.1)	0
Unknown	602 (83.5)	91 (100)
Outcome		
Died	130 (18.0)	18 (19.8)
Absent	18 (2.5)	0
LAMA	16 (2.2)	0
Discharged	557 (77.3)	73 (80.2)
Result for JEV laboratory test		
Positive	3 (0.4)	0
Negative	128 (17.8)	0
Awaited†	590 (81.8)	91 (100)

*Based on data obtained from Baba Raghav Das Medical College, Gorakhpur, Uttar Pradesh, India. JEV, Japanese encephalitis virus; LAMA, left against medical advice. †Clinical samples awaiting laboratory test results.

Using 2011 population data (*20*), we estimated that there were 20.2 AES cases/100,000 population in Kushinagar District in 2011 (Table 2). The incidence was higher among male residents than female residents (incidence rate ratio 1.29, 95% CI 1.11–1.49), and it was highest among children 0–6 years of age. The crude incidence rate ratio, comparing case-patients 0–6 years of age with those >6 years of age, was 7.97 (95% CI 6.87–9.25). Boys 0–6 years of age were at highest risk for AES. The incidence among 0- to 6-year-old boys was almost 50% greater than that among girls of the same age.

**Table 2:** Incidence of acute encephalitis syndrome, Kushinagar District, Uttar Pradesh, India, 2011–2012*

Age, sex of population	2011 population†	No. cases	Incidence‡	Incidence rate ratio (95% CI)
All ages	3,560,830	721	20.2	
M	1,821,242	414	22.7	1.29 (1.11–1.49)
F	1,739,588	307	17.6	1.0
0–6 y	551,467	428	77.6	
M	287,672	260	90.4	1.42 (1.17–1.72)
F	263,795	168	63.7	1.0

*Based on data obtained from Baba Raghav Das Medical College, Gorakhpur, Uttar Pradesh, India. †From 2011 Census of India (*20*). ‡Per 100,000 population.

**Figure 2:**
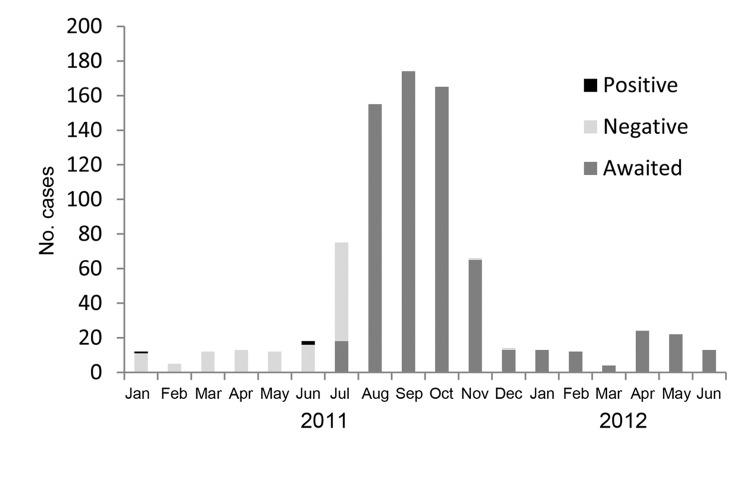
Weekly number of acute encephalitis syndrome cases, by month, in Kushinagar District, Uttar Pradesh State, India, 2011–2012. Numbers represent results of laboratory testing for Japanese encephalitis and are based on data from Baba Raghav Das Medical College, Gorakhpur, India. In the key, “awaited” refers to samples that were awaiting laboratory test results.

The weekly numbers of AES cases, classified by JE IgM laboratory result (positive, negative, awaiting determination), is shown in Figure 2. Only 3 (4.2%) cases of JEV infection were identified in 2011: two cases were in 55-year-old men, 1 of whom died, and 1 case was in a 14-year-old girl. Vaccination status was reported for 119 case-patients, of whom 3 (2.6%) had been vaccinated. The case-patients who had received vaccine were boys 6, 7, and 8 years of age; they began experiencing symptoms in July 2011 and were discharged within 3 weeks of hospital admission. None of the 3 JE case-patients had been vaccinated.

### Quality of Surveillance Data 

During data cleaning, we modified 25% of the 2011 and 5% of the 2012 line list values for residential locality, age, and date parameters (Table 3). Nearly one fifth of the age data and more than one fourth of the dates were edited. For 3.2% and 13.2% of cases in 2011 and 2012, respectively, the block name could not be determined from the residential locality provided and was marked as “unknown” because the village or police station name was not found or was present in multiple blocks. In addition, several fields in the database were incomplete. As of July 2012, laboratory results for JEV infection were still classified as “awaited” (i.e., awaiting determination) in the line lists for 82% (590) of the 721 cases in 2011 and for all 91 cases in 2012 (Table 1). The line lists indicated the date of sample collection, but the type of sample collected (CSF and/or serum) and the laboratory test used (IgM ELISA, PCR, and/or cell culture) were not recorded for any of the case-patients. Most samples submitted after July 2011 were still awaiting laboratory results at the time of our study (Figure 2). In addition, for 602 (83.5%) of the 721 case-patients in 2011 and all 91 case-patients in 2012, JEV vaccination status was marked as “unknown” in the line lists (Table 1).

**Table 3:** Values modified in line lists of case-patient data used in a study of acute encephalitis syndrome, Kushinagar District, Uttar Pradesh, India, 2011–2012*

Case-patient value	No. (%) modified		
	2011, n = 721	2012, n = 91	Combined, n = 812
Name of block of residence	192 (26.6)	2 (2.2)	194 (23.9)
Age	128 (17.8)	18 (19.8)	146 (18.0)
Date of symptom onset	221 (30.7)	4 (4.4)	225 (27.7)
Date of fever onset	213 (29.5)	4 (4.4)	217 (26.7)
Date of admission	193 (26.8)	2 (2.2)	195 (24.0)
Date of sample collection	186 (25.8)	1 (1.1)	187 (23.0)
Out come	139 (19.3)	2 (2.2)	141 (17.4
All fields	183 (25.4)	33 (36.3)	216 (26.6)

*Based on data obtained from Baba Raghav Das Medical College, Gorakhpur, Uttar Pradesh, India.

Key epidemiologic and programmatic indicators, such as the time between key points in AES disease progression and diagnosis, varied widely among case-patients (Table 4). In some instances, the dates were illogical (e.g., dates of symptom onset and sample collection following and preceding the dates of hospital admission, respectively). It was not possible to rectify these inconsistencies on the basis of the available data. In addition, the range of values was often large. For example, median time from onset of symptoms to hospital admission was 4 days in 2012, but some patients were admitted >2 months after symptom onset. Because AES is an acute syndrome, a long interval is not expected between symptoms and may indicate reporting of unrelated symptoms or misclassification of AES.

**Table 4:** Time between key points in disease progression and diagnosis for case-patients with acute encephalitis syndrome, Kushinagar District, Uttar Pradesh, India, 2011–2012*

Key points	Median time, d, between key points (range)	
	2011	2012
Symptom onset to hospital admission	7 (−4 to 39)	4 (1 to 63)
Symptom onset to sample collection	8 (1 to 40)	6 (1 to 64)
Hospital admission to sample collection	1 (−5 to 31)	1 (0 to 10)
Hospital admission to discharge or death	8 (0 to 70)	4 (1 to 17)

*Based on data obtained from Baba Raghav Das Medical College, Gorakhpur, Uttar Pradesh, India.

## Discussion

Despite the introduction of JE vaccine, an increased number of AES cases have been reported in Kushinagar District in recent years, and AES has been reported in areas not previously associated with the disease (*12**,**13*). Because of this apparently changing epidemiology of AES/JE in Kushinagar District, the importance of quality surveillance for guiding local and stratified caseload predictions for patient management and decision-making at policy and program levels cannot be overemphasized. However, our analyses show that despite recent attention regarding AES and public health interventions, constraints imposed by the district’s surveillance capacity hinder patient management, policy decisions, and implementation of prevention and control measures. The surveillance line list from BRD Medical College is representative of the district data on AES/JE (district malaria officer, pers. comm.). However, the current surveillance system captures only data for case-patients/samples that have been admitted/referred to BRD Medical College; thus, AES patients from Kushinagar District who seek medical care in the private sector are not represented in the surveillance data.

It seems that the surveillance database is not currently used for analysis at the district level; instead, the database is maintained only for administrative purposes, which include reporting to the State Directorate. However, the number of modifications that we had to make to the AES line lists for our analyses indicates that the quality of data collection is poor. We corrected some errors by making comparisons with other fields in the database, but other entries remain illogical (e.g., dates of hospital admission that proceed the dates of symptoms onset). Despite our thorough review and use of standardized protocol for cleaning the line lists, the results presented here should be viewed with regard to the inconsistencies in the original surveillance data. Regardless of the practical difficulties in coordination, collation, recording, and reporting of data, the high level of incomplete records, especially for JEV vaccination status and laboratory result, suggests a lack of initiative by data collectors to record complete and accurate information.

Current surveillance data provide little credible information to guide program planning and policy making for AES/JE in Kushinagar District. We could not determine if JE is etiologically responsible for AES in this area because reporting for JE laboratory testing was vague and incomplete. In addition, it is likely that only serum samples were collected to determine if AES was caused by JEV infection because the sampling procedure for serum is simpler than that for CSF. However, serum is a suboptimal sample for determining the cause of AES. Many JE infections are asymptomatic, so AES may be caused by an agent other than JEV even if JEV–specific IgM is present in the serum (*2**,**3*). In addition, a live, attenuated JEV vaccine is used in India, so the presence of JEV IgM in serum may be the effect of previous vaccination. CSF is preferred over serum samples for JEV testing because the presence of JE antibody in CSF provides a definitive diagnosis of JEV infection. A record of the type of sample collected is also essential for assessing the diagnostic yield of the sample and determining whether the sample was collected at the appropriate time after symptom onset (*2*). This need for a complex diagnostic process may have contributed to the incompleteness of laboratory results.

Regardless of the type of sample collected, the recording of JEV laboratory test results was inconsistent during the latter half of 2011 and nonexistent during 2012, despite collection of clinical samples soon after hospital admission. It is unknown whether the delays were caused by laboratory constraints or miscommunication in reporting the results. However, lack of timeliness in reporting surveillance data hinders its utility for guiding interventions and responding to outbreaks. JEV laboratory test results were available only during the low-transmission period, thus excluding any analysis for peak-transmission periods. JEV vaccination has been variously reported at 52% (*22*) to >95% (district health authorities, pers. comm.). We could not use the current surveillance data to estimate or validate the reported coverage figures because most vaccination histories were unknown.

Even basic epidemiologic analyses of demographic characteristics cannot be confidently interpreted as true or simply as the results of poor data collection. In the absence of reliable data, technical discussions have been overshadowed by shifting hypotheses about the etiology and epidemiology of AES, but these discussions are without a strong evidence base. For example, recent debates centered on the role of pigs in JEV transmission, yet the debates lacked relevant data about pigs in the area. In a similar manner, focus has shifted to waterborne causes of encephalitis, even though studies that have identified enteroviruses in patient samples have not concluded that waterborne pathogens are the main cause of AES incidence (*14*–*16*,*18*). These studies used different sampling methods and had different results, so the relative contributions of waterborne and vector-borne agents to AES cases in Kushinagar District remain unknown. Interventions cannot be planned when, depending on the etiology of AES, strategies as diverse as strengthening vaccination to improving water quality and sanitation may be appropriate. Quality surveillance data, including laboratory results and vaccination history, would resolve the inconsistencies between studies and inform intervention strategies.

## Conclusion

The current AES/JE surveillance system has a complicated specimen referral and reporting system at the district level, and the available line lists suggest that data are of low quality. Without evidence to estimate the effect of interventions, AES prevention and control measures may be ineffective and public health resources may be wasted. In 2011, AES and JE were highlighted in the national media, leading to a declaration for several policy initiatives, including formation of a multisectorial and interministerial National Encephalitis Control Programme (*23*). Despite the high profile of AES, the importance of surveillance data for guiding these initiatives has not been realized or translated to action. Gaps in surveillance capacity that were identified in this study indicate the need for a systematic evaluation of the AES/JE surveillance system in Kushinagar District and constitute key lessons that need to be incorporated as strategic planning is undertaken for this new initiative.
